# Framework for parallelisation on big data

**DOI:** 10.1371/journal.pone.0214044

**Published:** 2019-05-23

**Authors:** Lukman Ab. Rahim, Krishna Mohan Kudiri, Shiladitya Bahattacharjee

**Affiliations:** High Performance Cloud Computing Center, Universiti Teknologi PETRONAS, Seri Iskandar, Perak, Malaysia; Pablo de Olavide University, SPAIN

## Abstract

The parallelisation of big data is emerging as an important framework for large-scale parallel data applications such as seismic data processing. The field of seismic data is so large or complex that traditional data processing software is incapable of dealing with it. For example, the implementation of parallel processing in seismic applications to improve the processing speed is complex in nature. To overcome this issue, a simple technique which that helps provide parallel processing for big data applications such as seismic algorithms is needed. In our framework, we used the Apache Hadoop with its MapReduce function. All experiments were conducted on the RedHat CentOS platform. Finally, we studied the bottlenecks and improved the overall performance of the system for seismic algorithms (stochastic inversion).

## Introduction

Currently, oil and gas companies use seismic exploration technology to find oil and gas. Seismic data is big data that can be terabytes in size. A major problem in seismic data processing is the processing time. Many researchers [1, 2, 3] have used parallel processing techniques (for big data) to reduce the processing time. However, the processing technology for seismic data is different than normal big data processing technologies. Traditional data processing techniques are not suitable to deal with the high density of seismic data. For parallel processing, seismic attribute data are needed due to the pre-processing of input seismic data. The seismic attributes reflect the whole nature of the seismic data from different angles. With the continuous development of seismic attribute technology, the types of seismic attributes are increasing, and these attributes are applied to oil and gas exploration. Seismic attribute data not only help improve the utilization value of original seismic data, but also improve the application level of seismic technology in big data applications in the industry. In this research, seismic data (which are extracted as an attribute from original seismic data) will be saved into a file. Here, these seismic data, or attribute data, will be suitable for parallelisation using Hadoop technology.

Seismic attribute data are generally analysed using high performance parallel computing methods. Currently, Chevron uses the Hadoop MapReduce approach for the analysis of seismic attribute data [4]. Nevertheless, Cloudera works with Hadoop for seismic processing [5]. However, seismic processing using big data technology is still in the exploratory stage. MapReduce was introduced by Google to provide distributed and parallel processing for many applications [6]. However, this technique is not suitable for small data. It is suitable for seismic data processing because it has large inputs and small processing techniques [7, 8]. Thus, the general objective of this research work is to create a template (Mapper and Reducer) for Hadoop that can provide parallelisation for any seismic algorithms through seismic attribute data (which is created from actual seismic data). Hadoop is a very simple but powerful and efficient computing framework. The idea behind Hadoop is that instead of moving the data to the computations, we move the computations to the data [9]. In the older days, people used simple algorithms on big data to improve the system execution time. However, Hadoop allows us to apply more complex algorithms on the required data, which improves not only the system’s execution time but also its accuracy.

**Fig 1:**
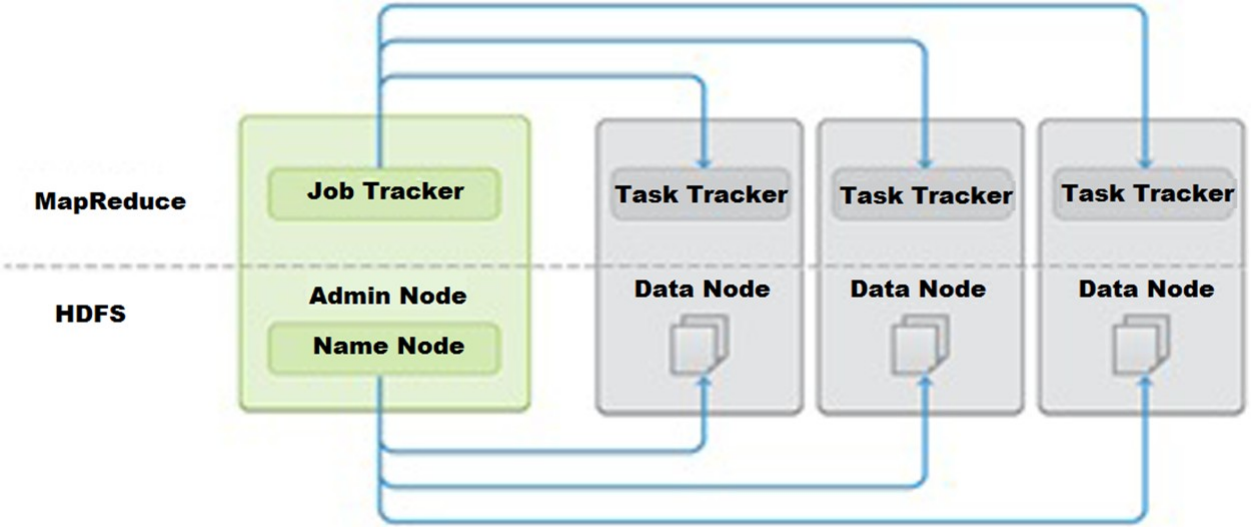
Hadoop architecture [13].

The two main basic components of Hadoop are the Hadoop Distributed File System (HDFS) and Hadoop MapReduce [10, 11]. The Hadoop framework itself is mostly written in the Java programming language. It has some applications in native C and the command line utilities that are written in shell scripts. HDFS is a Hadoop database can store large files, which are typically in the range of gigabytes, terabytes, and petabytes, across multiple machines [11, 12]. Hadoop MapReduce is our parallel processing framework that will map and reduce data. If we talk about Hadoop as an overall architecture, it consists of four parts, namely, the NameNode, DataNode, TaskTracker, and JobTracker, which are illustrated in “Fig 1”. A typical MapReduce engine consists of a JobTracker in which client applications can submit MapReduce jobs, and this JobTracker typically pushes work out to all the available TaskTrackers.

The MapReduce framework is essentially the software’s framework. It was originally developed for parallel processing applications under Hadoop [12, 13, 14, 15, 16, 17]. The basic idea is that the job splits the data into chunks, and these chunks are processed by the mapper by laying them out across all the computing nodes. Once the map tasks are done processing these individual and independent chunks of data, the framework sorts the output from this process. The reduced task will use this sorted map data as its input and perform some reduction operations on them to produce the output for the entire programme. All applications that were developed in the original Hadoop framework use this approach.

The advantage is that the computing and storage nodes are typically the same. Thus, one’s map tasks and HDFS demands are part of the data nodes, which are essentially running on the same nodes. This means that one can schedule tasks on nodes that already have data. This is important if one is looking for scaling because he is not moving data between nodes, but actually computing where the data are.

In this case, the mapper finds the maximum arrival delay in each chunk of data. The mapper then stores these maximum values as the intermediate values associated with the key. The reducer receives a list of maximum arrival delays for each chunk and finds the overall maximum arrival delay from the list of values. The MapReduce only calls this reducer once, since the mapper only adds a single unique key. The reducer uses it to add a final key-value pair to the output.

The rest of this report is as follows. Sections 1 and 2 will respectively present the introduction and methodology of the proposed technique. Section 2 will also discuss the basic concept of Hadoop. In the same section, the said technique is proposed and designed for solving, addressing, and resolving the processing time issue. Section 3 presents the complete implementation of the said technique with examples. Section 4 justifies the performance and efficiency of the said technique. Finally, Sections 5 concludes and gives suggestions for future work.

## Methodology

**Fig 2:**
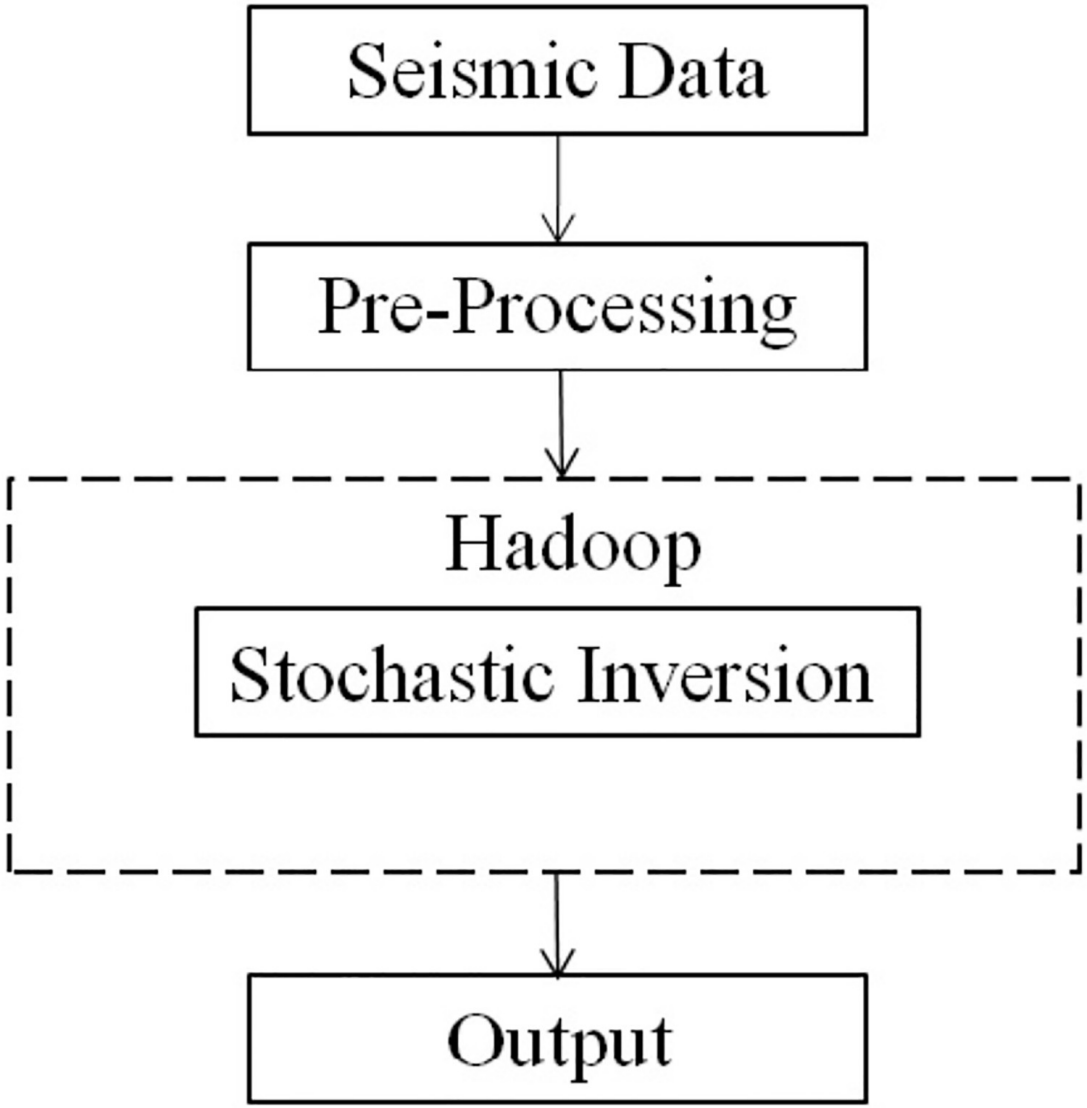
Schema of the work.

Seismic data come from a band limited signal. To acquire seismic reflection data, the frequency band needs to be extended. Stochastic inversion addresses this problem by generating a range of plausible solutions that can then be narrowed through testing for the best fit against various measurements. Current techniques have high processing times when executing the seismic algorithm (stochastic inversion). Thus, in this research work, we used a wrapper for the seismic algorithm (stochastic Inversion) in Apache Hadoop, as illustrated in “Fig 2”.

The use of the Map/Reduce framework (which is part of Hadoop) will help us make a wrapper for big data processing (seismic data), but we have to adopt the Map/Reduce requirements. In other words, Map/Reduce expects us to write certain kinds of functions in exchange for taking care of the logistics. The first requirement is that all our data are going to be placed into key-value pairs. We could think of it as a key-value pair that is going to become our basic unit of data and our unit of analysis. The other requirement is that the user has to specify the mapper and reducer functions. The mapper is the function that is applied to the data, while the reducer is the function that is applied to the intermediate results that come from Hadoop. The mapper is a user-defined function, and that function will read in the data and output a key-value pair. However, the user also defines the reducer function, which is built to read in the key-value pairs and output a result. Hadoop handles all the logistics of the parallel execution of the map, including reducing the functions, producing intermediate results, and communicating those results to the reducers.

**Fig 3:**
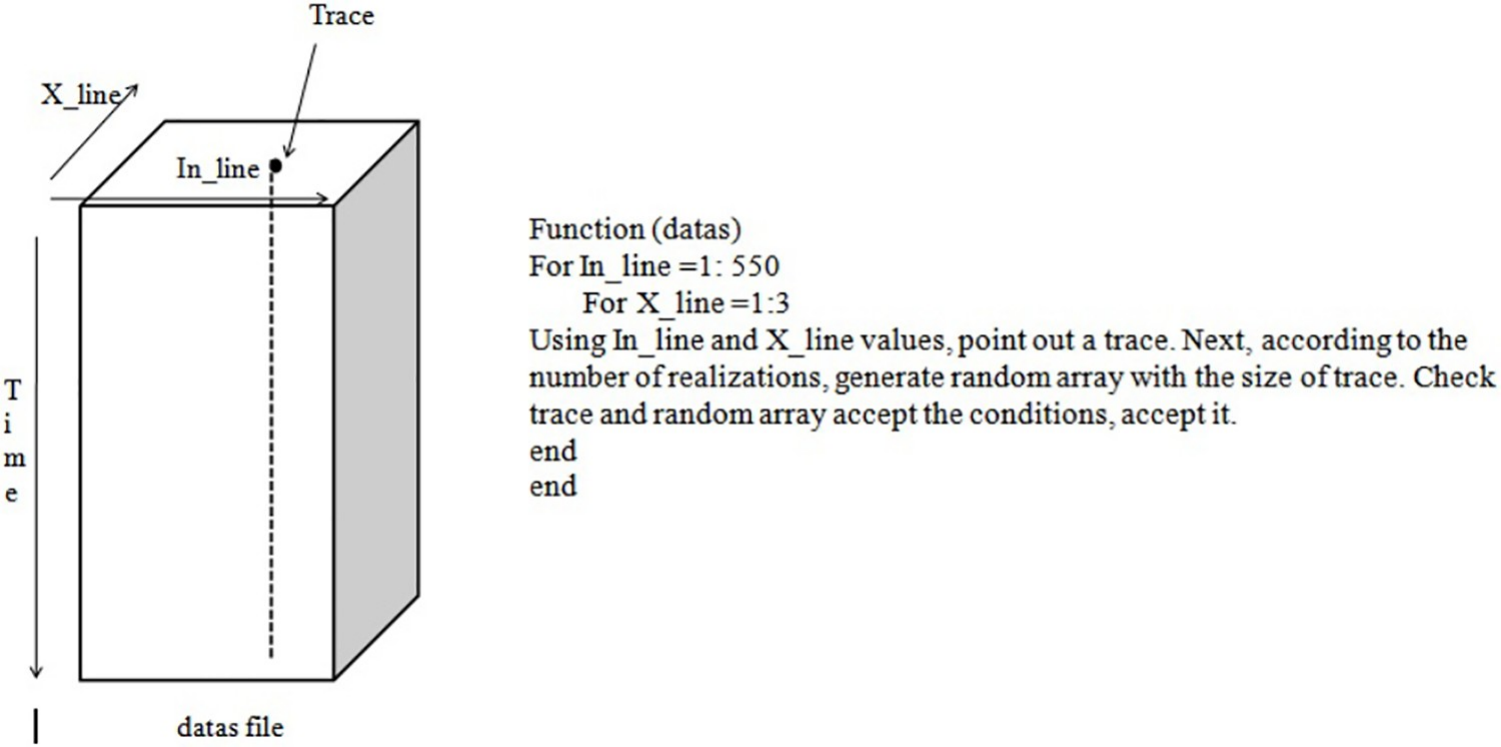
Stochastic inversion algorithm for seismic data.

The implementation of the mapper function for stochastic inversion is as follows. This mapper function reads each trace from the input segy file and executes 10 realisations for each trace. Here, data come from Hadoop HDFS. This mapper function is executed on each block of data and each block comes back as data. Thus, each mapper function reads the block of data and computes its sum and length. Finally, the reducer function collects all the above sub sums and computes the total average for the given input. For instance, in this research work, we worked on a stochastic inversion algorithm. In this case, the input is a 3D file containing large field data (12 km X 11 km) and is named as “datas”, as illustrated in “Fig 3”.

Here, the MapReduce function with the location of a trace point through the X_line and In_line values is applied. According to the location of the trace point, the MapReduce function decides how to send the trace data to the mapper. Once the mapper function receives the data, it computes and sends it to the Reducer as a list of key-value pairs. In this case, the key will be the location of the data (X_line, In_line and Time), while the value will be the trace data. The Reducer receives the data and combines them into a single unit.

**Fig 4:**
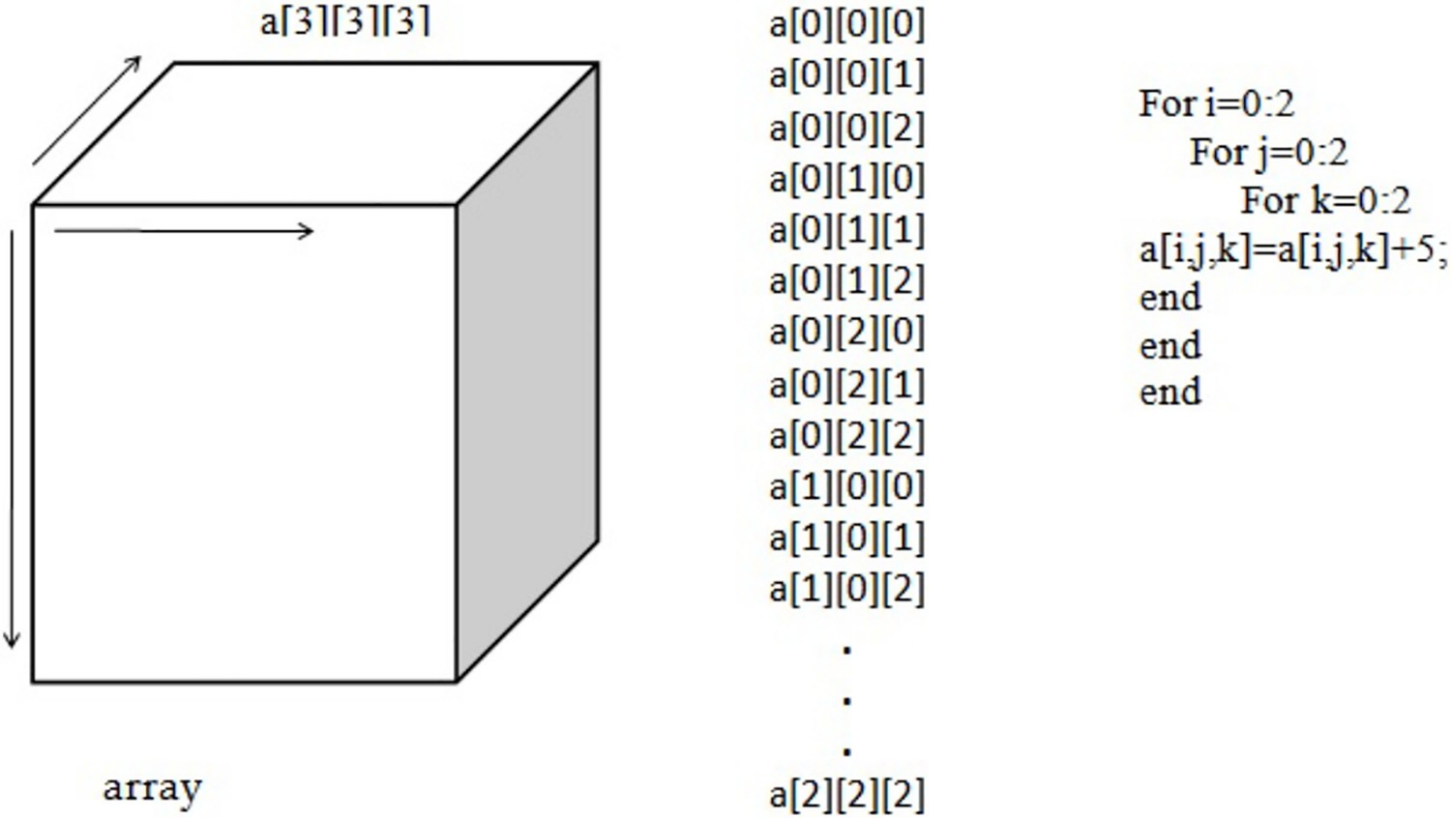
Three-dimensional array.

The function to process “datas”, is similar to a 3D array, which is illustrated below in “Fig 4”.

In this example, “a” is an array of size 3 x 3 x 3. The total number of elements or locations in this array is 27. To access a subset of this array, we need three loops, each of size 3. There are always two ways to process a data-set. One is selecting the columns in data-set, which are also called as the Z-axis in data file. The other way is by selecting the rows, which are also called the X-axis in given data file.

**Fig 5:**
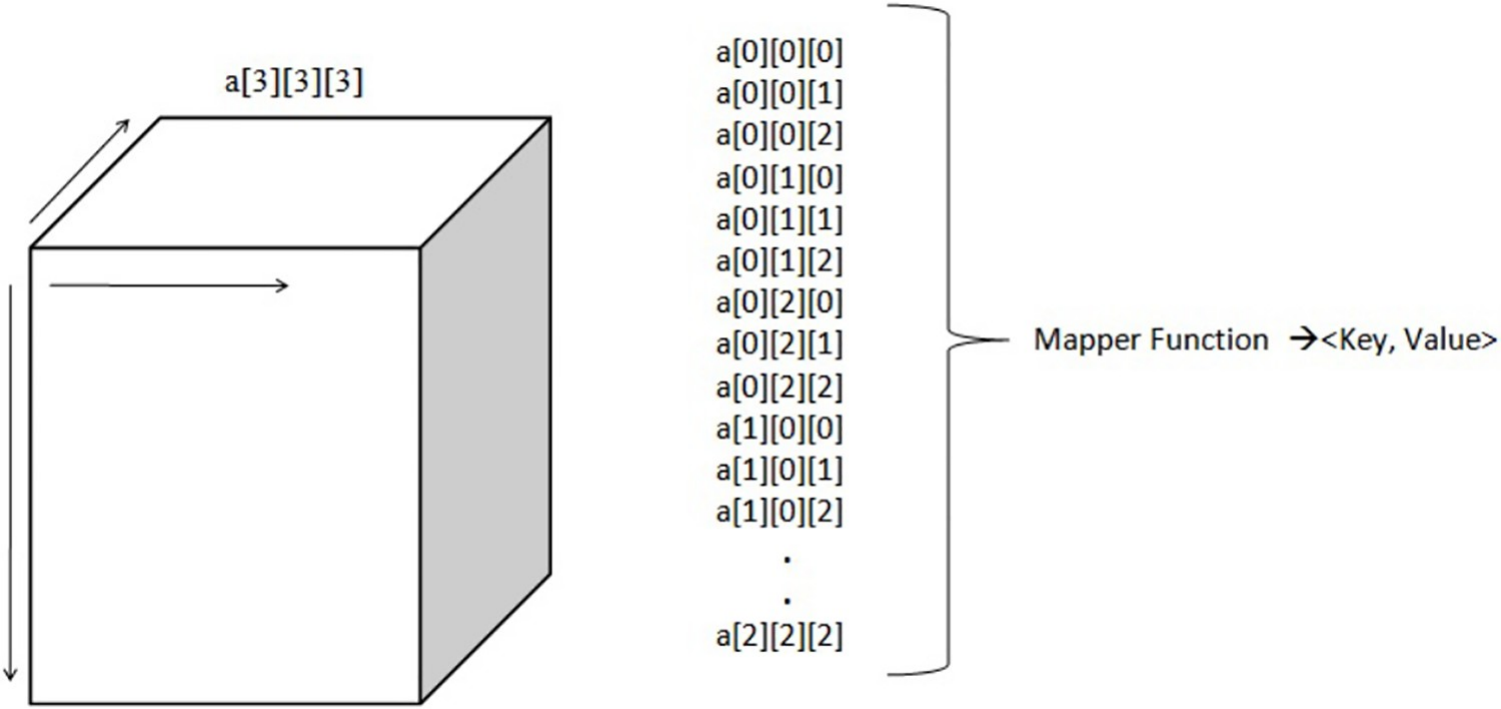
Key and value pairs for a three-dimensional array.

In this example, we can access the subset of arrays in the form of rows. However, these types of methods are only applicable for smaller databases. If the database is so big that we cannot even store it in a single computer, Hadoop then comes into the picture. Hadoop helps save data on data clusters and processes it using its mapper and reducer functions. In this example, the mapper function performs the selection of not only the row but also the locations of it. This approach assumes that the data-set can fit in the memory after the mapper phase. Here, the mapper receives chunks of data and outputs intermediate results. The reducer reads the intermediate results and produces the final result. Let us consider the same data array “a” but with a bigger size. Each location of the database acts as an input to the mapper function and produces the key and value pairs for the reducer function, as illustrated in “Fig 5”.

**Fig 6:**
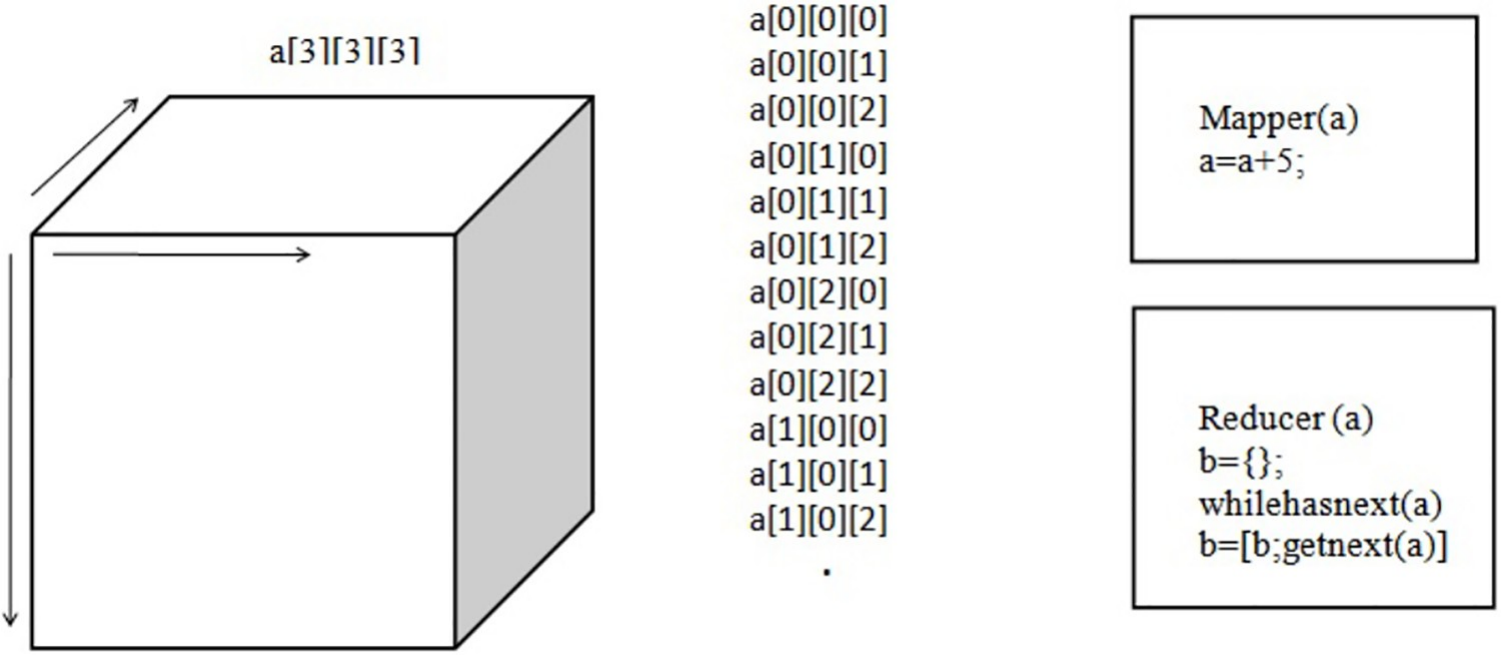
Mapper and reducer functions for each location.

**Fig 7:**
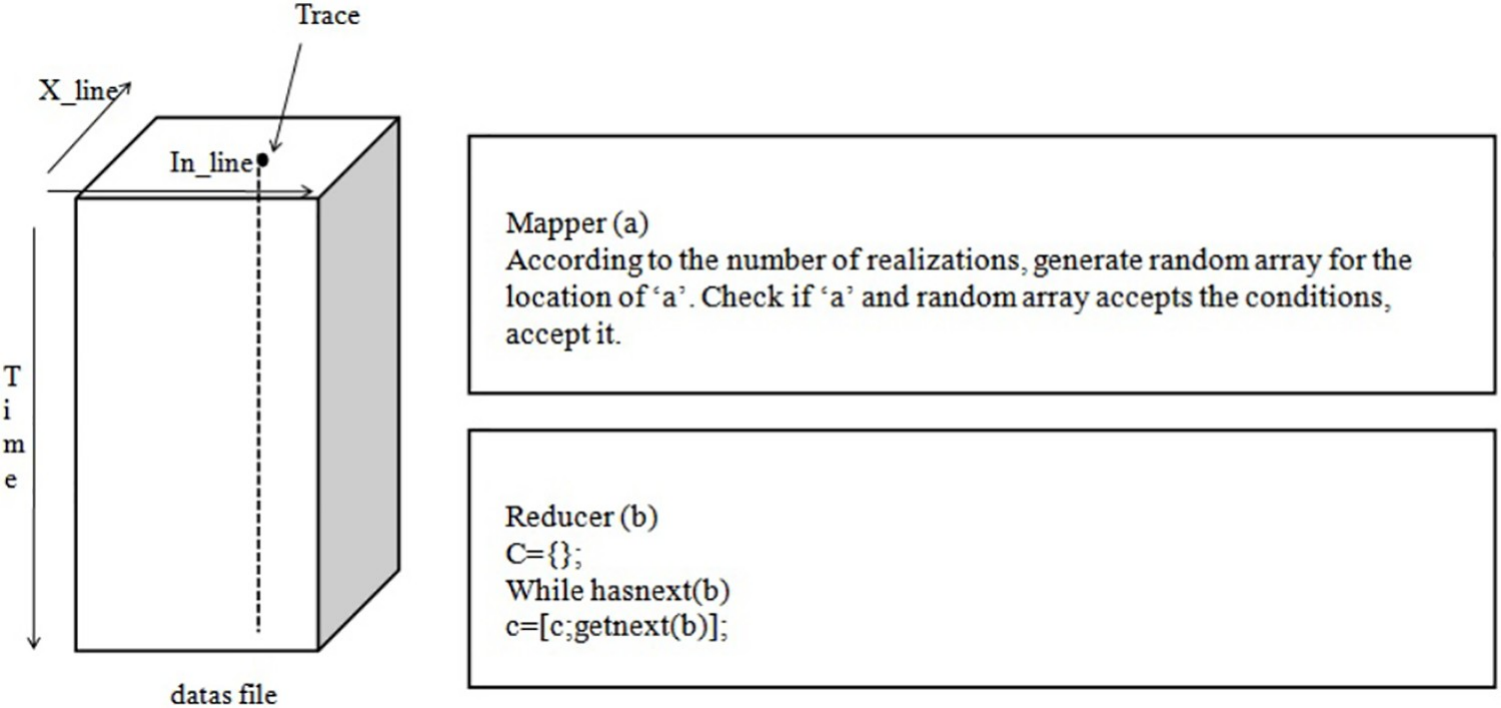
Mapper and Reducer function for stochstic inversion algorithm.

The mapper and reducer for this given example are illustrated in “Fig 6”. Here, the mapper receives each location as an input and produces an output. Similarly, the reducer receives all the outputs from the mapper and combines them. In this process, these mapper and reducer functions are completely focused on every location of the input data-set. Depending on the application, we can also create a mapper function that can process a selected set of rows (X-axis) or columns (Z-axis) of the given input database. “Fig 7” shows how the mapper and reducer functions were implemented for the stochastic inversion problem. In this case, the processing of each trace, which is also called a column (Z-axis), is important. This is because when we get the location of a particular trace from the loop function, we need to use that location in order to compute its number of realizations.

Here, we call the MapReduce Function with the location of a trace point through the X_line and In_line values. According to the location of the trace point, the MapReduce function decides how to send the trace data to the Mapper. Once the Mapper function receives the data, it computes and sends the output to the Reducer as a list of key value pairs. In this case, the key will be the location of the data (X_line, In_line and Time). The value will be the trace data. The reducer receives the data and combines them into a single unit.

## Experimental setup

In this experiment, we installed the Apache Hadoop-2.7.1 on the CentOS 6 platform. We implemented it on a total of four node clusters, including NameNode. Before the Hadoop installation, we installed Java JDK on the Hadoop cluster. We also installed MATLAB-Runtime to the cluster to use direct MATLAB executable files. In this evaluation process, the experimental setup for the following experiment is illustrated in Table 1. Table 2 further illustrates the list of parameters for the experiments. Additionally, the database for small and large field data are illustrated in Tables 3 and 4, respectively. Here, the NameNode is connected to three DataNodes. The tuning parameters for the following experiment are illustrated in Table 5 below.

**Table 1:** Hadoop cluster configuration (Hitachi Servers).

Configuration (Hitachi Servers)
CPU: Linux 2.6.32 (CentOS 6.4). Totally 16 core (32 hyper thread)
Memory: 128 GB
OS: Linux 2.6.32 (CentOS 6.4)
Platform: Hadoop

**Table 2:** Parameters for the experiment.

Evaluation Parameters			
Sampling rate (in/out)	2 msec/ 2 msec	Frequency	0-90Hz
Number of samples	20	Number of realizations	10
Constrain Number	3	Noise	3
Minimum Correlation	0.1		

**Table 3:** Small field data.

Profile of Small Field Data	
Area	unknown
Area Size	5.5km X 0.03km (3D)
Number of Trace Inline	Inline:550, xline:3
Interval of Trace	10m

**Table 4:** Large field data-set.

Profile of Large Field Data	
Area	Kumang, Malaysia
Area Size	11km X 12km (3D)
Number of Trace Inline	Inline:1100, xline:1200
Interval of Trace	10m

**Table 5:** Tuning parameters for bottleneck analysis.

Tuning Parameters	
Data Block Size	64 MB (Default)
Number of OS threads	32 hyper thread
Number of Data Nodes	3 (Excluding Name Node)
Number of Map Tasks	2 (Default)

In this section, bottleneck analysis to improve the processing time was done using a list of measurements, which are the average CPU/MEMORY/IO usage in each node and the time series of the CPU/IO usage in each node.

## Results and discussion

**Fig 8:**
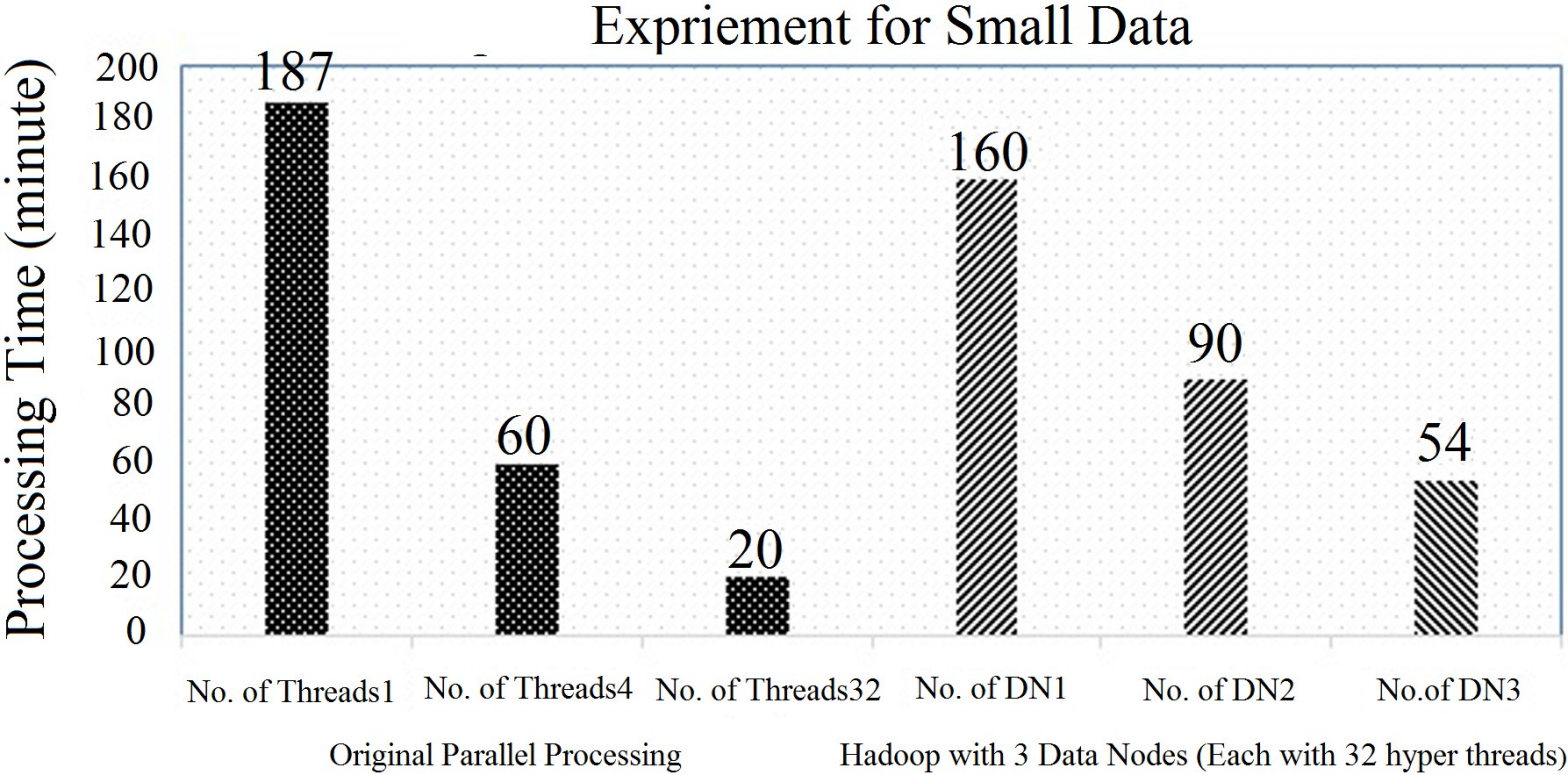
Comparison of original parallel processing with Hadoop (3 data node) with 32 hyper threads.

**Fig 9:**
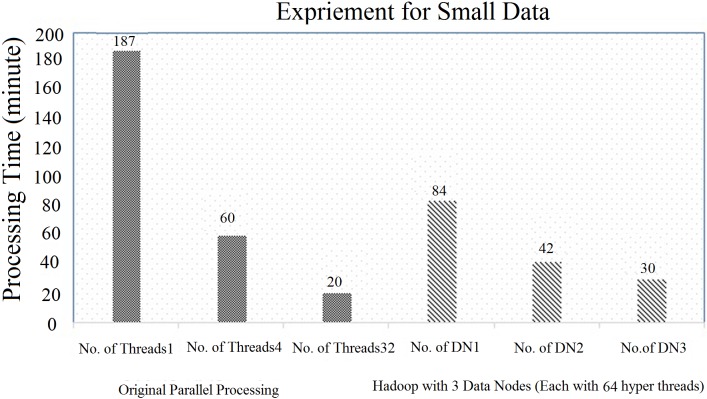
Comparison of original parallel processing with Hadoop (3 data node) with 64 hyper threads.

In this section, an evaluation was done using a small data-set with Hadoop (with three DataNodes) from the original code, as illustrated in “Figs 8 and 9”. First, the original parallel processing was compared with the Hadoop with multiple data nodes (DN), such as DN1, DN2 and DN3. The original parallel processing for a single thread, 4 threads and 32 threads used the execution times of 187 minutes, 60 minutes and 20 minutes, respectively. Similarly, Hadoop (with each node having 32 threads and with a block size of 64 MB) took 160 minutes (for DN1), 90 minutes (for DN2) and 54 minutes (for DN3), respectively, as illustrated in “Fig 8”. In this case, the performance of the original parallel processing was higher than Hadoop.

To counter the issue, the number of threads for each data node in Hadoop was increased from 32 hyper-threads to 64 hyper-threads, as illustrated in “Fig 9”. In this case, Hadoop (with each node having 64 threads and with a block size of 64 MB) took 84 minutes (for DN1), 42 minutes (for DN2) and 30 minutes (for DN3), respectively, which were better than Hadoop with 32 hyper threads (already discussed in “Fig 8”). However, in both cases, it was concluded that the performance of the original parallel processing was always higher due to the following reasons: 1) 100 percent usage of the CPU cores, and 2) the default block size of the original parallel processing was smaller than that of Hadoop. Thus, to counter the issue, multiple Data Nodes were added and the total number of hyper threads that was used was also increased. Finally, the Hadoop (default block size of 64 MB) was able to perform similarly to the original parallel processing technique, as illustrated in “Figs 8 and 9”.

**Fig 10:**
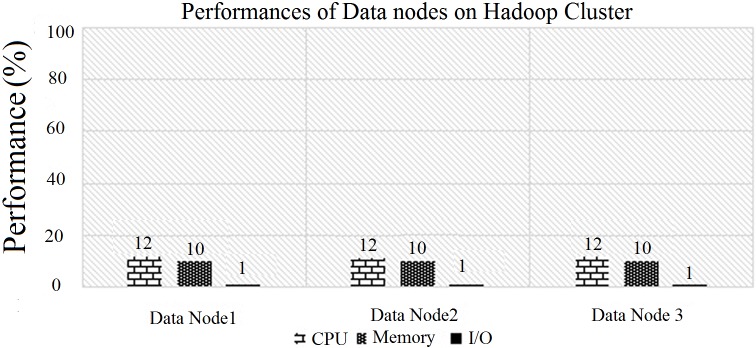
CPU, memory, and IO performances of Hadoop.

Next, the performance of each DataNode on the Hadoop cluster is illustrated in “Fig 10”. Here, the performances of the CPU usage, Memory, and IO is are almost the same for each DataNode on the Hadoop cluster.

**Fig 11:**
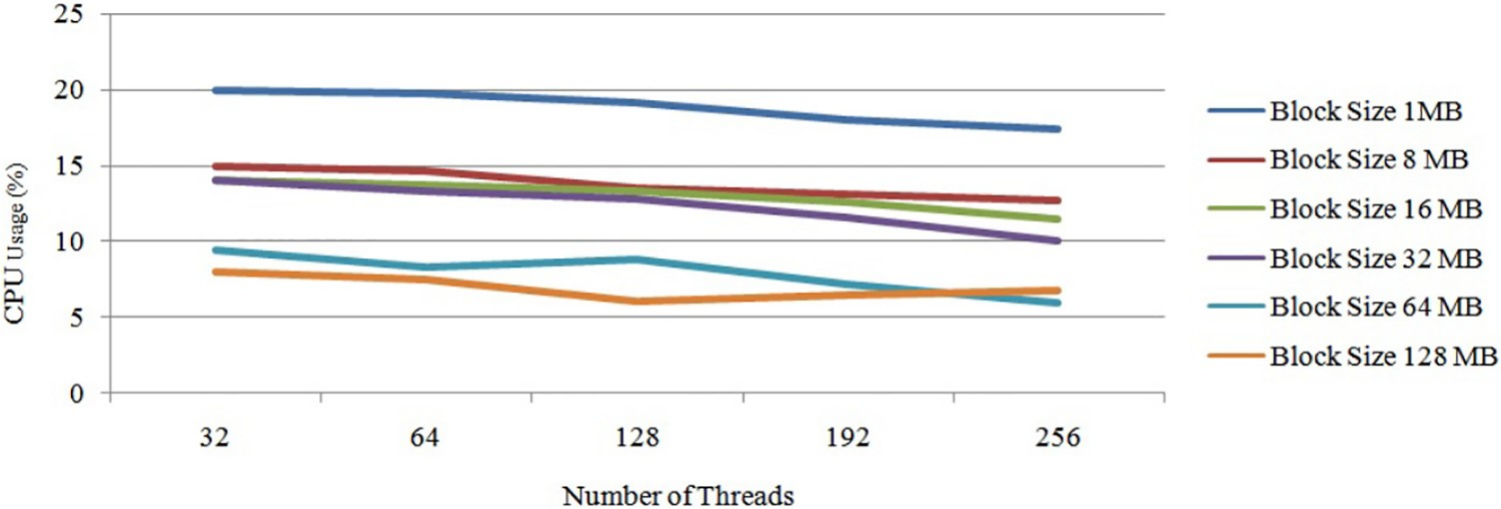
Performance of CPU threads at each DataNode on Hadoop cluster.

Next, “Fig 11” illustrates the average performance of the OS threads in Hadoop with a single DataNode. In this experiment, we first chose a DataNode with 32 threads and conducted the experiment with different block sizes. Next, we chose the same DataNode with 64 threads and repeated the same experiment. According to the experiments, when we increased the number of OS threads, the average CPU usage was stable between 32 to 64 threads. When we increased the number of threads to more than 64, the CPU usage started to decrease due to the lack of parallelisation of the mapper tasks. Next, the average performance of threads is illustrated in “Fig 11”. In this cluster, the maximum CPU usage was 21 percent and the minimum was 5 percent. The average CPU usage was 12 percent.

**Fig 12:**
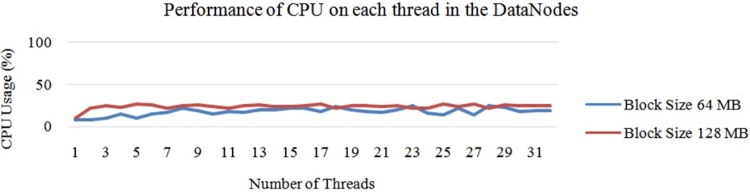
Average usage of Hadoop on large data-set with different block sizes.

From “Fig 11”, it can be concluded that the usage of the CPU hyper threads rises with a smaller block size. Additionally, it is understood that the usage of each CPU hyper thread decreases when we increase the possible number of hyper threads. These changes are due to the task tracker’s capacity of accepting the number of mapper tasks at once. Next, according to “Fig 11”, it can also be concluded that the number of CPU hyper threads were inversely proportional to the usage of the average CPU core. This is due to two issues: 1) the lack of parallelisation, and 2) the size of the data-set. To overcome this issue, we can fix the number of mapper tasks for the task tracker. Similarly, increasing the size of the input data-set also helps increase the usage of the CPU, as illustrated in “Fig 12”.

**Fig 13:**
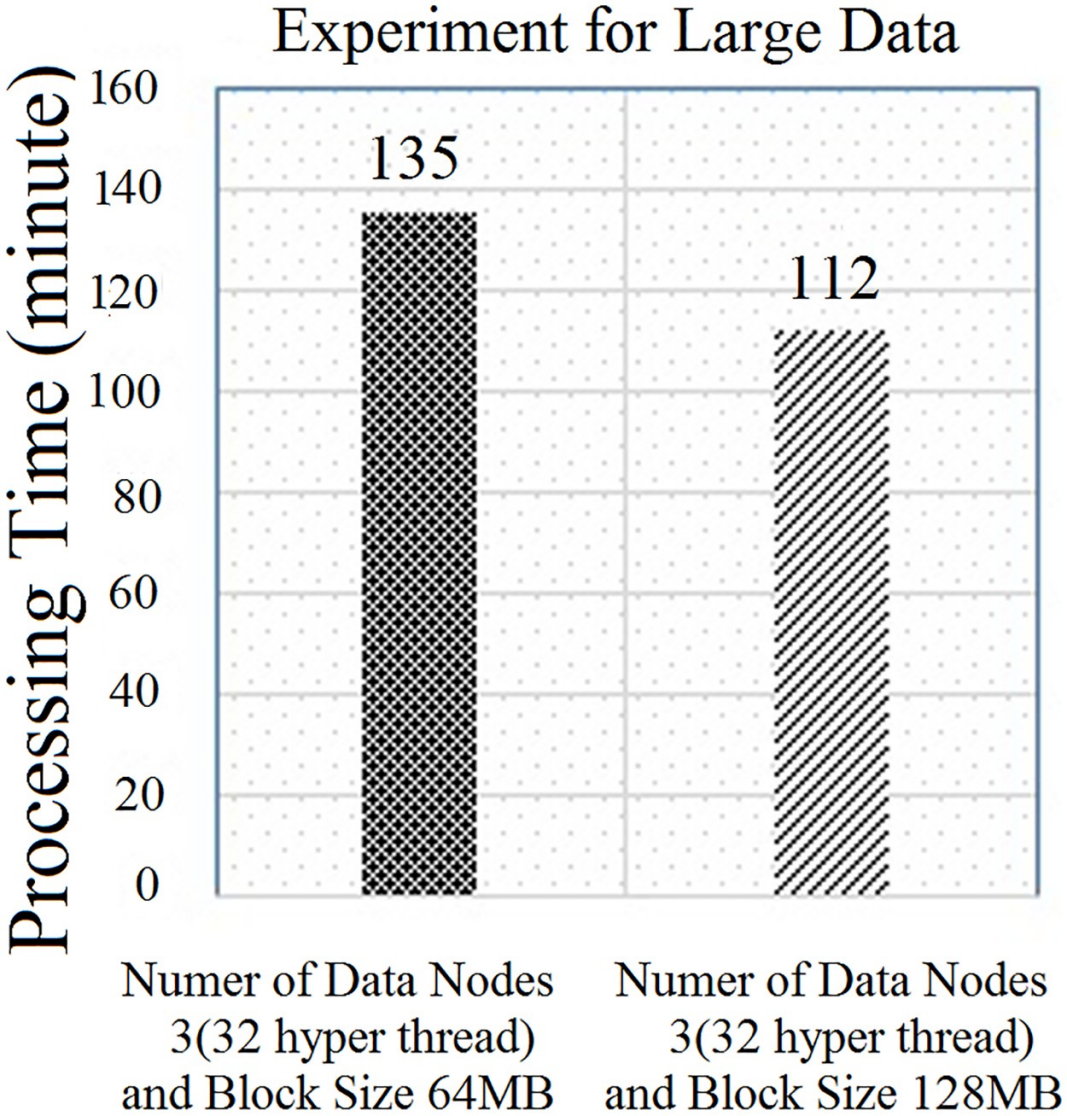
Comparison of Hadoop processing time on large data with different block sizes.

**Fig 14:**
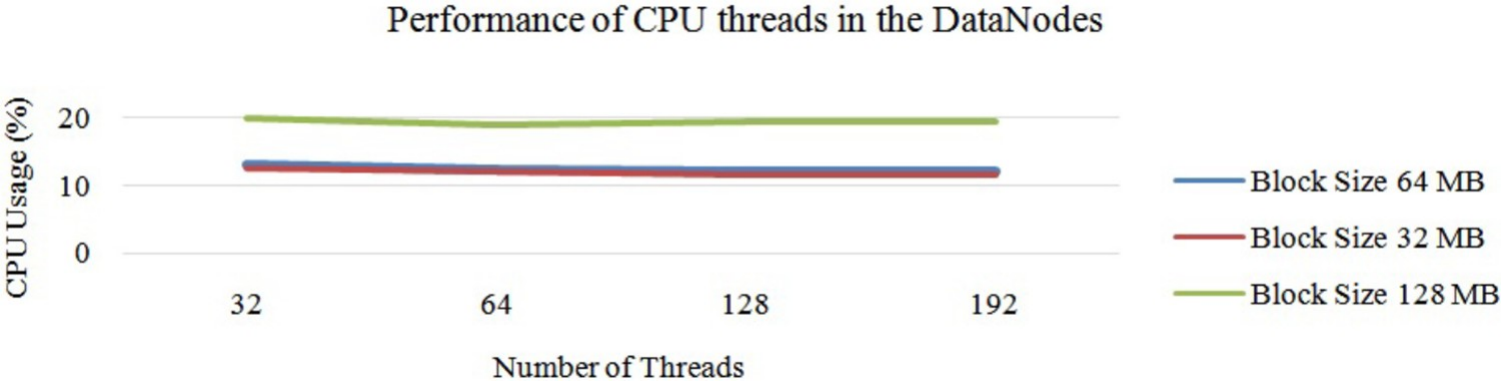
Performance of Hadoop on each DataNode for large data with different block sizes.

“Fig 12” shows the experimental results for a larger data-set. According to “Fig 12”, it can be concluded that the CPU usage is higher for larger block sizes. “Fig 13” shows the experimental results for a large field data (12 km x 11 km). In this case, the size of the input data-set is 1.5 GB. In this experiment, the execution time of each mapper is less than 30 seconds, which has already been discussed in Section 3. Due to that, the CPU usage is very minimal. To counter the problem, we have two options as follows: firstly, first tune the number of map tasks to the task tracker, and, second, increase the block size. In this experiment, which is illustrated in “Fig 13”, we increased the block size from 64 MB to 128 MB as illustrated in “Fig 13”. When we increased the block size in the HDFS, the CPU utilization was raised. Due to this situation, the processing time also decreased compared to the actual default block size, which is illustrated in “Fig 14”. In this case, the block size of 128 MB showed the optimum CPU usage compared to the rest. To overcome this limitation, we needed to tune the number of map tasks to the task tracker.

**Fig 15:**
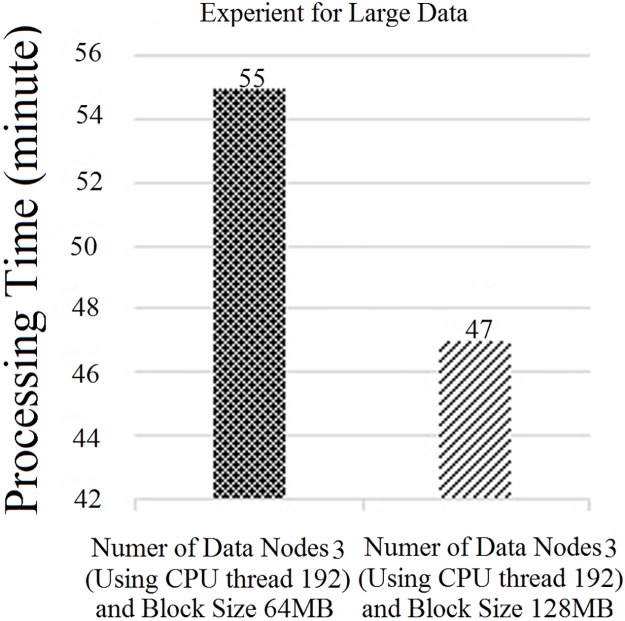
Performance of Hadoop (64 threads each DataNode) on large data with different block sizes.

**Fig 16:**
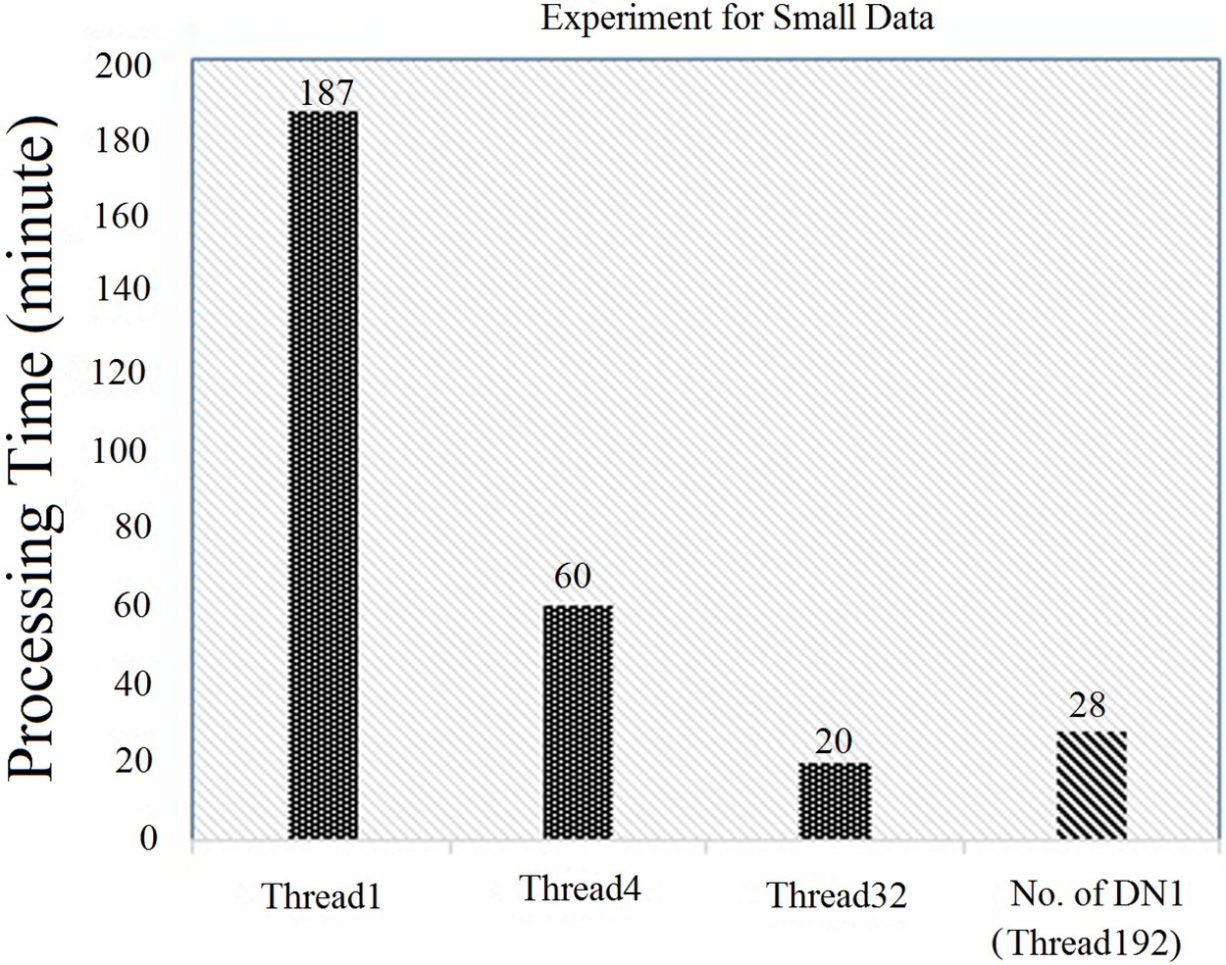
Experiment using single DataNode with 192 threads.

“Fig 15” shows the average CPU usage for different block sizes using Hadoop with a single node. Based on “Fig 15”, it can be concluded that the average CPU usage decreases slowly when we increase the number of CPU hyper threads. However, the CPU usage for 64 hyper threads was similar to that with 32 hyper threads. Nevertheless, bigger block sizes had higher CPU usage than the smaller block sizes on the larger data-set due to the execution time of each task, which has already been discussed in Section 3. Thus, we increased the number of threads from 32 threads to 192 threads. However, the performance of the CPU thread was almost the same as that illustrated in “Fig 15”. In this case, the block size of 128 MB showed an optimum performance compared to the rest. Increasing the number of threads to more than 192 also decreases the CPU usage drastically. Here, 192 threads was the optimum number. Thus, the overall processing time had decreased, as illustrated in “Fig 16”.

From “Figs 15 and 16”, it can be concluded that in order to achieve the peak performance speed of the parallel processing technique, Hadoop needs to use threads with three DataNodes. However, the fact is that increasing the total number of nodes to optimize the execution speed is practically impossible for big data. Thus, in this experiment, which is illustrated in “Fig 16”, we raised the number of threads for the single node to achieve the peak performance speed of the parallel processing technique.

**Fig 17:**
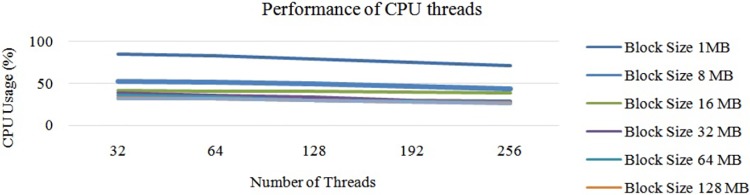
Performance of CPU threads at 2 map tasks allocated to each task at maximum on single DataNode.

**Fig 18:**
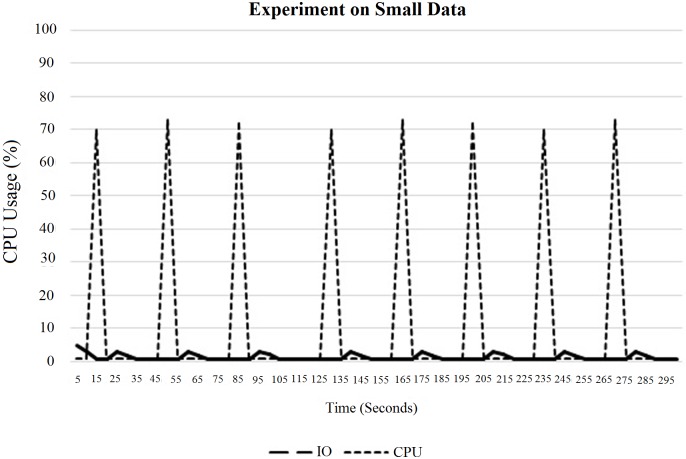
CPU and IO usage for a single DataNode using small data.

In fact, increasing the total number of threads is not a good idea for parallel processing because it may cause lack of parallelisation issues, which have already been discussed in Section 3. Here, in “Fig 17”, the CPU usage did not decrease due to the overhead from switching the OS thread. This instead had happened due to the small amount of inputs. Next, “Fig 18” shows the CPU and IO performance for a default block size of 64 MB.

According to “Figs 17 and 18”, it can be concluded that the performance of threads depends on the block size. In this case, a smaller block size has greater CPU usage than the larger one. However, in fact, a smaller block size increases the IO usage at the NameNode, which affects the overall performance of the system.

**Fig 19:**
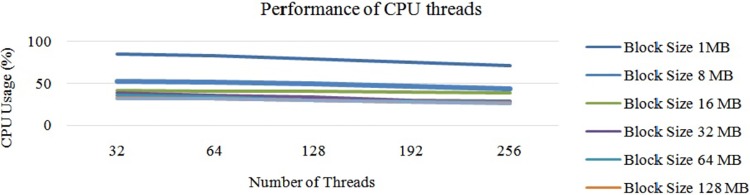
Maximum number of map tasks that should run simultaneously by a task tracker.

According to “Fig 15”, it can also be concluded that CPU threads are idle for a long time for the default block size of 64 MB. This is due to insufficient parallel tasks. Thus, we need to adjust the map tasks and reduce tasks in order to achieve proper parallelisation. Here, we tuned the maximum number of map tasks that should run simultaneously using a task tracker, as illustrated in “Fig 19”.

“Fig 15” shows the CPU and IO performance for tuned map tasks that should run simultaneously using a task tracker for a block size of 1 MB.

**Fig 20:**
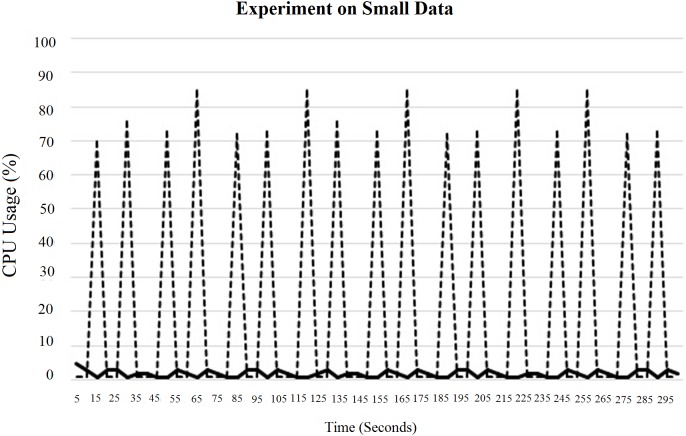
CPU usage with tuned map tasks for a single DataNode using small data.

In this case, we kept the maximum number of map tasks, that should run simultaneously using a task tracker, equal to the total number of threads used. This improved the usage of CPU threads and helped to improve the overall performance of the system, as illustrated in “Fig 20”.

**Fig 21:**
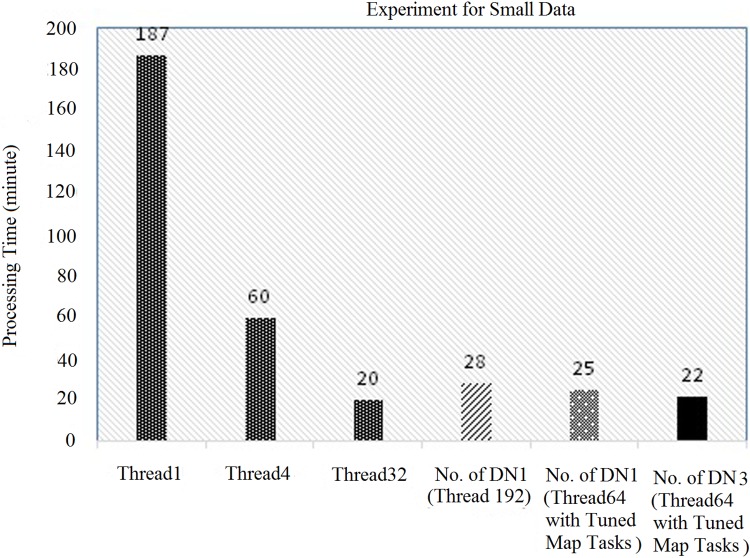
Experiment with tuned map tasks using single DataNode.

Next, we proceeded with the same experiment but with large data-sets, as illustrated in “Fig 21”. In this case, the larger block size performed better than the smaller block size. This was because the larger block size could handle more data and produce a smaller number of reducer tasks, which helped the Hadoop to provide parallelisation.

**Fig 22:**
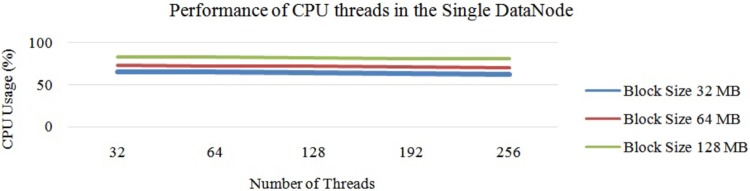
Performance of CPU threads for larger data.

**Fig 23:**
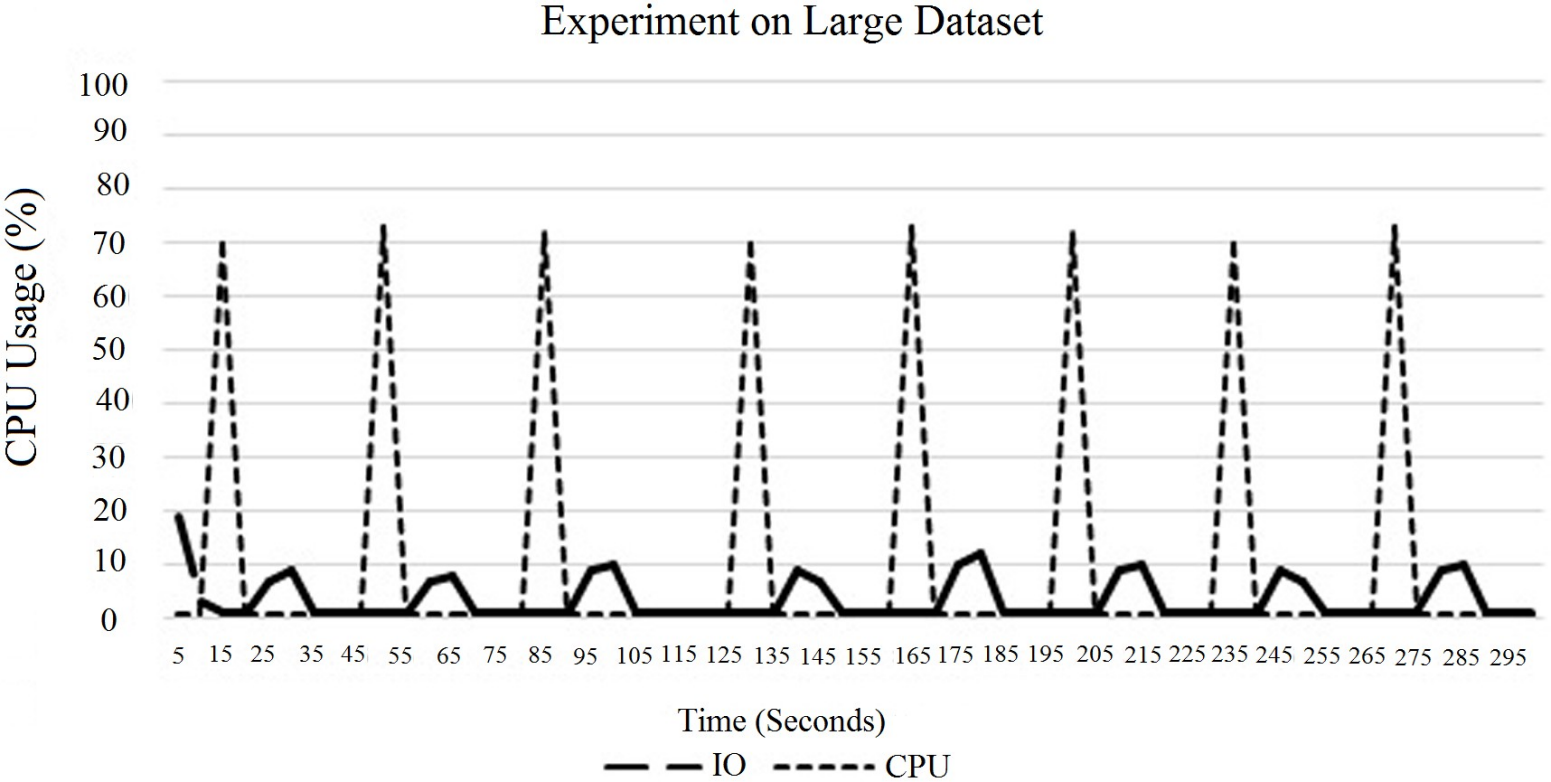
CPU and IO usage on a large data-set for a single DataNode.

**Fig 24:**
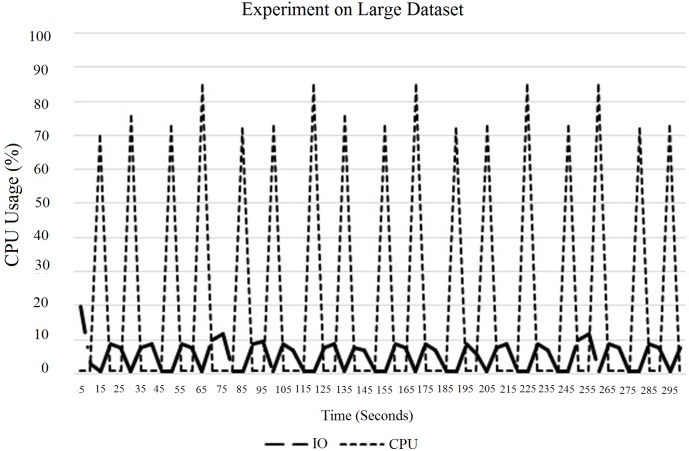
CPU and IO usage on large data-set for a single DataNode with tuned map tasks.

**Fig 25:**
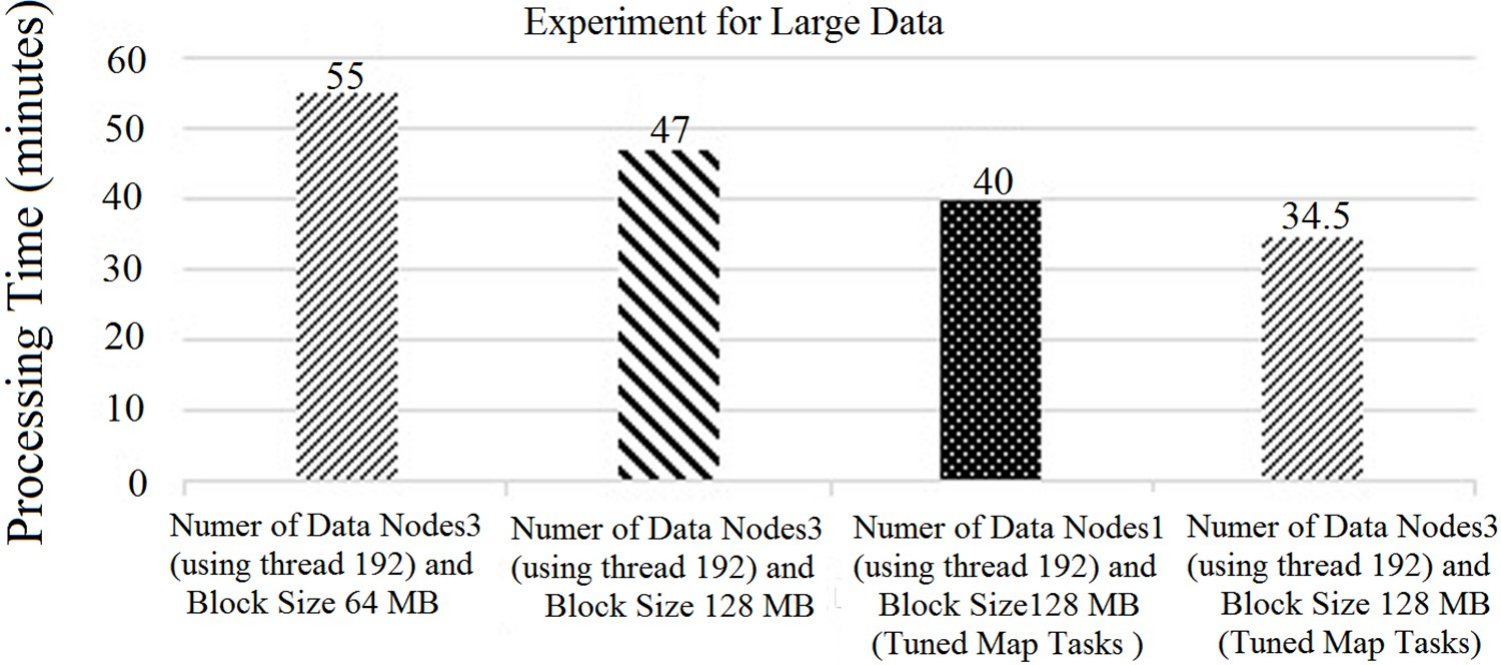
Experiment with tuned map tasks using single DataNode.

As shown in “Figs 22, 23, 24 and 25”, large data perform better with larger block sizes. In this case, we tuned the maximum number of map tasks that should run simultaneously using a task tracker at 192 threads because the performance of the system was better with 192 threads. Next, from “Figs 22 and 25”, it can be concluded that, depending on the size of the input data-set, the CPU usage changes. For instance, if the input data-set is smaller, then the block size should be smaller. Similarly, if the input data-set is bigger, then the bigger block size provides better CPU usage than the smaller ones. According to the overall experiment, it can be concluded that the performance of Hadoop depends on the size of the input data-set, the number of CPU hyper threads, the maximum number of map tasks that should run on a task tracker, and the block size.

## Conclusion and future work

The use of parallel processing in seismic applications is becoming popular with the improvements of processing speeds. However, when dealing with big data such as seismic algorithms, basic issues such as the processing speed become major issues. These complications increase drastically due to huge data sizes. Hence, in this research work, a wrapper or template is introduced to provide parallelisation for big data applications such as seismic algorithms using Hadoop. This technique is capable of providing parallelisation for big data such as megabytes, gigabytes, terabytes, petabytes, exabytes, and zetabytes of data in a very easy manner using the Hadoop map-reduce technique. Additionally, this wrapper can be implemented in C, C++, Java, MATLAB, and Python on the Hadoop platform.

In this research work, we evaluated and justified the performances of this technique. The performance produced by using the stochastic algorithm was measured by calculating its processing speed for different block sizes, different numbers of processes, and different DataNodes. In this experiment, the performance of a stochastic inversion was tested using both small and large data-sets. In this study, we found a bottleneck due to the IO wait, which caused a lack of parallel tasks on the Hadoop cluster. Due to that, the average utilization of CPU threads fell to 10 percent. However, the original parallel processing utilising the CPU core reached 100 percent and performed better than the Hadoop cluster with a single DataNode. To counter the issue, we adjusted the number of mapper and reducer tasks, the number of CPU hyper threads, and the block size. Finally, we achieved a higher performance with a 30X speed improvement with a single DataNode over the traditional parallel processing technique. It is concluded that the performance of Hadoop depends on the size of the input, the number of CPU threads used, the maximum number of map tasks for the job tracker, and the memory block size.

Normally, the possible bottlenecks for Hadoop are as follows: 1) massive IO caused by large input data in the map input stage, where; the solution is to compress the input Data; 2) massive IO caused by spilled records in the partition and sort phases, where; the solution was adjusted to spill records and sort the buffer formula for io.sort.mb; 3) massive network traffic caused by a large map output, where; the solution is to compress the map output; 4) massive network traffic caused by a large reduce output, where; the solution is to adjust the replication factor; and 5) insufficient parallel tasks, where; the solution is to adjust the number of map tasks, the number of reduce tasks, and amount of memory. Future research can work to improve this technique since it had failed to improve the speed performance with small data-sets compared to the traditional parallel processing technique. The Spark on Hadoop can be used in the proposed technique to improve the performance. As this research work had focused only on offline analysis, it is hoped that in the near future this work can be extended for real-time analysis using the Spark.

## Supporting information

S1 File Datas. This is a 3D input file containing large field data (12 km X 11 km).
(ZIP)
